# Radiographic outcomes and non-union factor analysis in fragmentary segmental femoral shaft fractures (AO/OTA 32C3) treated with reamed antegrade nailing

**DOI:** 10.1038/s41598-024-59136-x

**Published:** 2024-04-10

**Authors:** Won-Tae Cho, Jae Hoon Jang, Seung Ryeol Park, Hoon-Sang Sohn

**Affiliations:** 1 Department of Orthopaedic Surgery, School of Medicine, Ajou University Hospital, Suwon, Republic of Korea; 2 Department of Orthopaedic Surgery, Dong-eui Hospital, Busan, Republic of Korea; 3 Department of Orthopaedic Surgery, Wonju Severance Christian Hospital, Yonsei University Wonju College of Medicine, 162 Ilsan-dong, Wonju, 26426 Republic of Korea

**Keywords:** Exposed nail length, Femur shaft fracture, Femoral shaft fragmentary segmental fracture, Non-union, Health care, Medical research, Risk factors

## Abstract

This study retrospectively assessed radiographic outcomes and risk factors associated with non-union in femoral shaft fragmentary segmental fractures (AO/OTA 32C3) treated with reamed antegrade intra-medullary nailing. Radiological outcomes, including union and alignment, were evaluated. The risk factors for non-union were investigated, including demographics and treatment-related characteristics, such as the number of interlocking screws, segmentation length, main third fragment length, distance of the main third fragment, width ratio and exposed nail length in one cortex from immediate post-operative radiographs. Multivariate logistic regression was used for statistical analysis. Among 2295 femoral shaft fracture patients from three level-1 trauma centers, 51 met the inclusion criteria. The radiological union was achieved in 37 patients (73%) with a mean union time of 10.7 ± 4.8 months. The acceptable axial alignment was observed in 30 patients (59%). Multiple logistic regression analysis identified only exposed nail length as a significant risk factor for non-union (odds ratio: 1.599, *p* = 0.003) and the cut-off value was 19.1 mm (sensitivity, 0.786; specificity, 0.811). The study revealed high rates of non-union (27%) and malalignment (41%). Therefore, patients who underwent intramedullary nailing with an exposed nail length greater than 19.1 mm or about twice the nail diameter should be cautioned of the potential non-union.

## Introduction

Femoral shaft fragmentary segmental fractures (AO/OTA classification 32C3) are uncommon and result from high-energy injuries that are frequently associated with life-threatening conditions^1^. According to the AO/OTA classification, segmental fractures of the long bone diaphysis are divided into intact segmental and fragmentary segmental fractures^2^. Fragmentary segmental fractures of the femur shaft are challenging to surgically treat in terms of restoring functional alignment and reducing fragments. Therefore, high rates of several complications, such as limb shortening, malalignment, infections and non-union, have been reported^3^.

There are several treatment options for femoral shaft fracture. However, intra-medullary nailing is considered the gold standard treatment^4^. Several studies have reported favourable outcomes for femoral shaft fractures treated with intra-medullary nailing^5^ and risk factors for non-union have been evaluated^6–11^. However, no study has evaluated the outcomes of femoral shaft fragmentary segmental fractures owing to their low incidence (approximately 5–7% of femoral shaft fractures)^1,12,13^. Furthermore, no study has reported on the factors affecting non-union in these fractures.

Therefore, this study aimed to assess the radiographic outcomes of intra-medullary nailing for femoral shaft fragmentary segmental fractures and evaluate the risk factors associated with non-union.

## Methods

### Ethical approval

All experimental protocols were approved by Wonju Severance Christian Hospital, Yonsei University Wonju College of Medicine, the Institutional Review Board (IRB). All procedures and methods performed in the studies involving human participants were in accordance with the ethical standards of the institution or practice at which the studies were conducted (IRB number: CR323033). The requirement for informed consent was waived owing to the observational nature of this study.

### Patient selection

**Figure 1:**
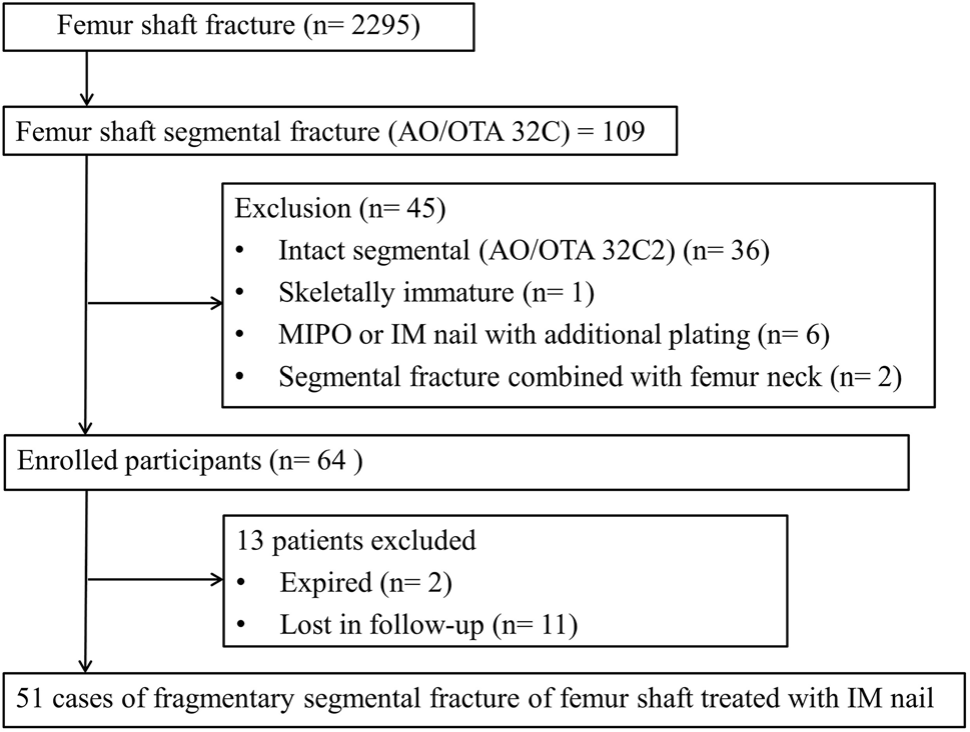
Patient flowchart.

This was a multi-center (three level-1 trauma centres) retrospective study between December 2012 and January 2020 for eligible patients. The inclusion criteria were skeletally mature fractures, acute femoral shaft fragmentary segmental fractures (AO/OTA 32C3) and patients treated with femoral intra-medullary nailing. The exclusion criteria were treatment with additional plates, follow-up for > 12 months and pathologic fractures. Of 2295 femoral shaft fractures screened, 51 met the criteria and were included (Fig. 1).

### Demographic and radiological assessment

The assessment variables could be divided into demographic and injury characteristics, and treatment-related characteristics.

Demographic and injury characteristics included age; sex; body mass index (BMI); smoking status; diabetes status; injury mechanism; polytrauma; open fracture; fracture location (AO/OTA 32C3(i), proximal diaphyseal–metaphyseal fracture; 32C3(j), pure diaphyseal fracture; 32C3(k), distal diaphyseal–metaphyseal fracture)^14^; and number of fragments in the segment. A fragment larger than 40 mm was considered capable of affecting fracture healing^15^.

**Figure 2:**
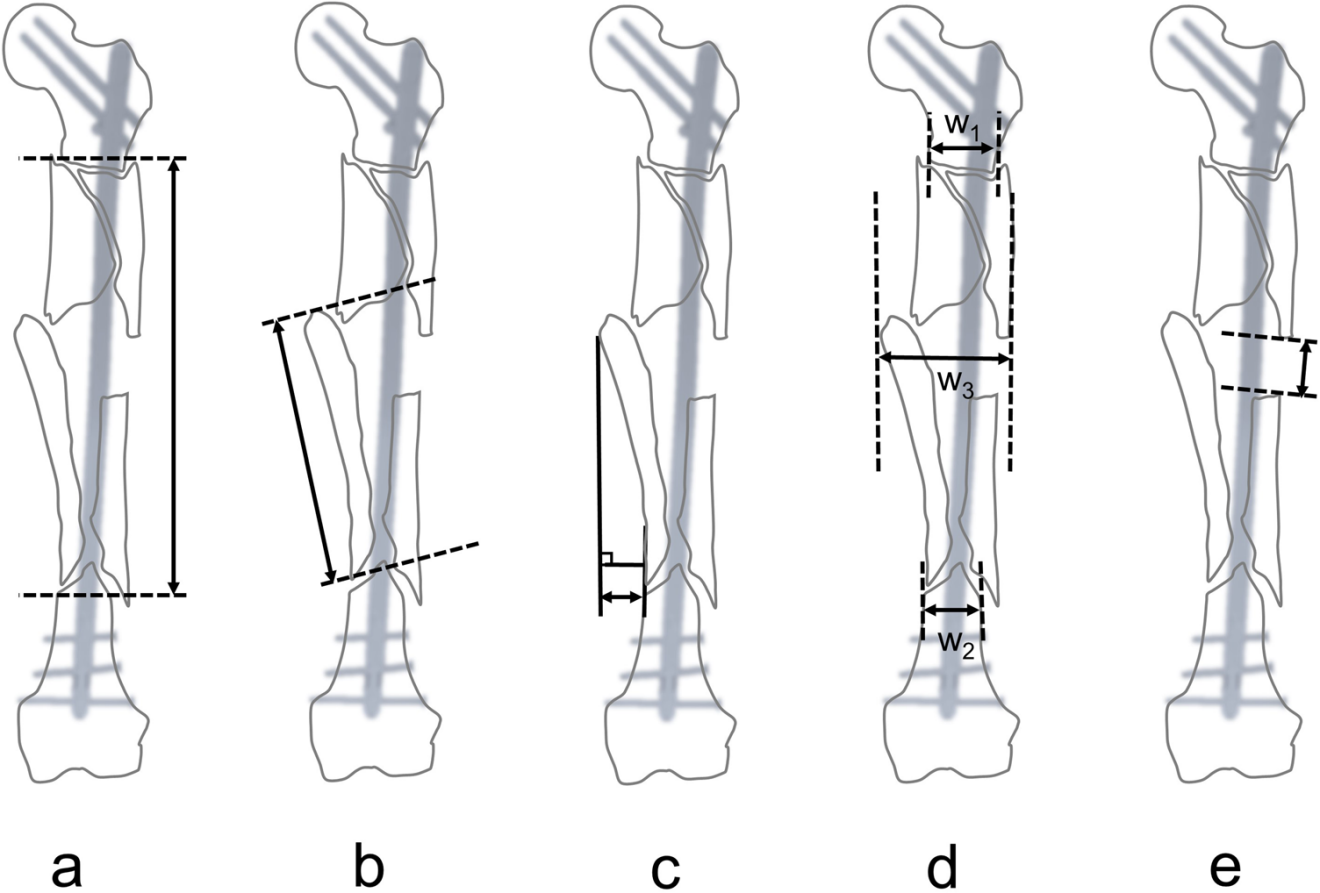
(**a**) The segmentation length was defined as the length of the fragmentation between the intact proximal and distal fragment. (**b**) The main third fragment length was defined as the length of the long axis of the largest fragment. (**c**) The distance of the main third fragment was defined as the longest perpendicular distance between the main third fragment and the nearest intact cortex of the shaft. (**d**) The width ratio was defined as the ratio of the maximum fragmentary width (w_3_) to the mean diameter of the fracture site (mean diameter of the fracture site = [w_1_ + w_2_]/2). (**e**) The exposed nail length was defined as the longest length of the exposed nail parallel to the nail axis in one cortex from immediate post-operative radiographs.

Treatment-related characteristics included the number of interlocking screws in the proximal and distal fragments, segmentation length, main third fragment length, distance of the main third fragment, width ratio, and exposed nail length from immediate post-operative radiographs (Fig. 2).

The fracture union rate, mean union time and modified Radiological Union Scale for Tibia (mRUST) at months 1–3 and then every 3 months after surgery were assessed until complete healing or non-union. The application of this scoring system to femoral shaft fractures is valid and reliable^16,17^. The scoring criteria were as follows: 1 point, clear fracture line between the fracture ends; 2 points, callus between the fracture ends, but the fracture line remains visible; 3 points, bridging callus formation between the fracture ends, with a fracture line; and 4 points, bone bridge formation between the fracture ends, without a fracture line. A score of 16 in four cortices in the anteroposterior and lateral views suggested complete fracture healing. Fracture union was defined as the presence of a bridging callus in at least three cortices (mRUST score ≥ 13). Non-union was defined as the absence of evidence of the healing process in radiographs over 3 months or fractured bones that had not completely healed within 9 months^18,19^. In a scanogram, the alignment of the coronal and sagittal planes, as well as the length of the limbs were evaluated. An acceptable alignment was considered if there was less than or equal to 5° of coronal and sagittal plane angular deformity. An axial malalignment was defined as more than 5° of angulation, including varus or valgus deformity and flexion or extension deformity. Furthermore, limb length discrepancy was defined as more than a 15 mm difference, which could be either shortening or lengthening compared to the opposite side of the limb^20–22^.

Two orthopaedic surgeons, who were blinded to patient characteristics, retrospectively reviewed the medical records and radiographs twice, 1 month apart. For radiological parameters, the average measurements of the two observers were calculated and used. Moreover, both anteroposterior and lateral radiographs were used, and the largest value was considered. Disagreements were resolved through discussion.

### Surgical treatment

All patients underwent reamed antegrade intra-medullary nailing through closed reduction or percutaneous reduction techniques, including cerclage wiring, joystick manoeuvre using a Hohmann retractor or Schanz pin, and a blocking screw or pin. The surgeon determined the type and number of interlocking screws according to their preference and fracture configuration. The Expert Asian Femoral Nail (A2FN; Synthes, Solothurn, Switzerland), Femoral Recon Nail (FRN; Synthes, Solothurn, Switzerland) and Zimmer Natural Nail (ZNN; Zimmer, Warsaw, IN, USA) were the titanium femoral nails used in all patients.

### Rehabilitation

After surgery, active range of motion exercise and quadriceps muscle strengthening were initiated at the knee and hip joints. Patients started partial weight bearing with walking aids on post-operative day 3. Depending on tolerance, the exercise intensity and duration were gradually increased. Full weight bearing was permitted 4 weeks after the operation.

### Statistical analysis

Continuous variables were analysed using the independent sample *t*-test or Mann–Whitney *U* test, and the chi-square or Fisher’s exact test was used for categorical variables to compare demographic data and clinical outcomes between the two groups. Univariate logistic regression was used to analyse all variables. The variables with a *p*-value of < 0.15 were analysed using multivariate logistic regression analysis. Moreover, no significant multicollinearity of the variables was found when defining occurrence as a variance inflation factor of > 10 (data not shown). Cohen’s kappa coefficient was used to assess both intra-observer reliability and inter-observer agreement of radiographic measurements. Receiver operating characteristic curves were created to determine the cut-off values of significant variables for predicting non-union risk, and optimal cut-off values that maximised the sum of sensitivity and specificity were determined. A *p*-value of < 0.05 was considered statistically significant. All statistical analyses were performed using SPSS software (version 25.0; SPSS Inc., Chicago, IL, USA), with a 95% confidence interval.

## Results

The study included 51 patients (43 [84%] males and 8 [16%] females; mean age, 40.1 ± 13.7 years). The mean follow-up period was 36.8 ± 17.0 months, and the mean time to surgery was 5.45 ± 4.0 days. A2FN was used in 27 patients (52.9%), FRN in 13 patients (25.5%) and ZNN in 11 patients (21.6%). There were 20 smokers (39.2%) and 7 patients (13.7%) on diabetic medication, including insulin injections. Open fractures were diagnosed in 7 patients (13.7%) (classification: Gustilo–Anderson type I, 4 patients; II, 2; IIIa, 1). Open wounds were treated with debridement and primary wound repair or skin grafting. Moreover, 43 patients (84.3%) had polytrauma with concomitant injuries, in chest, abdomen and brain, which required additional treatment and hospitalisation.

**Figure 3:**
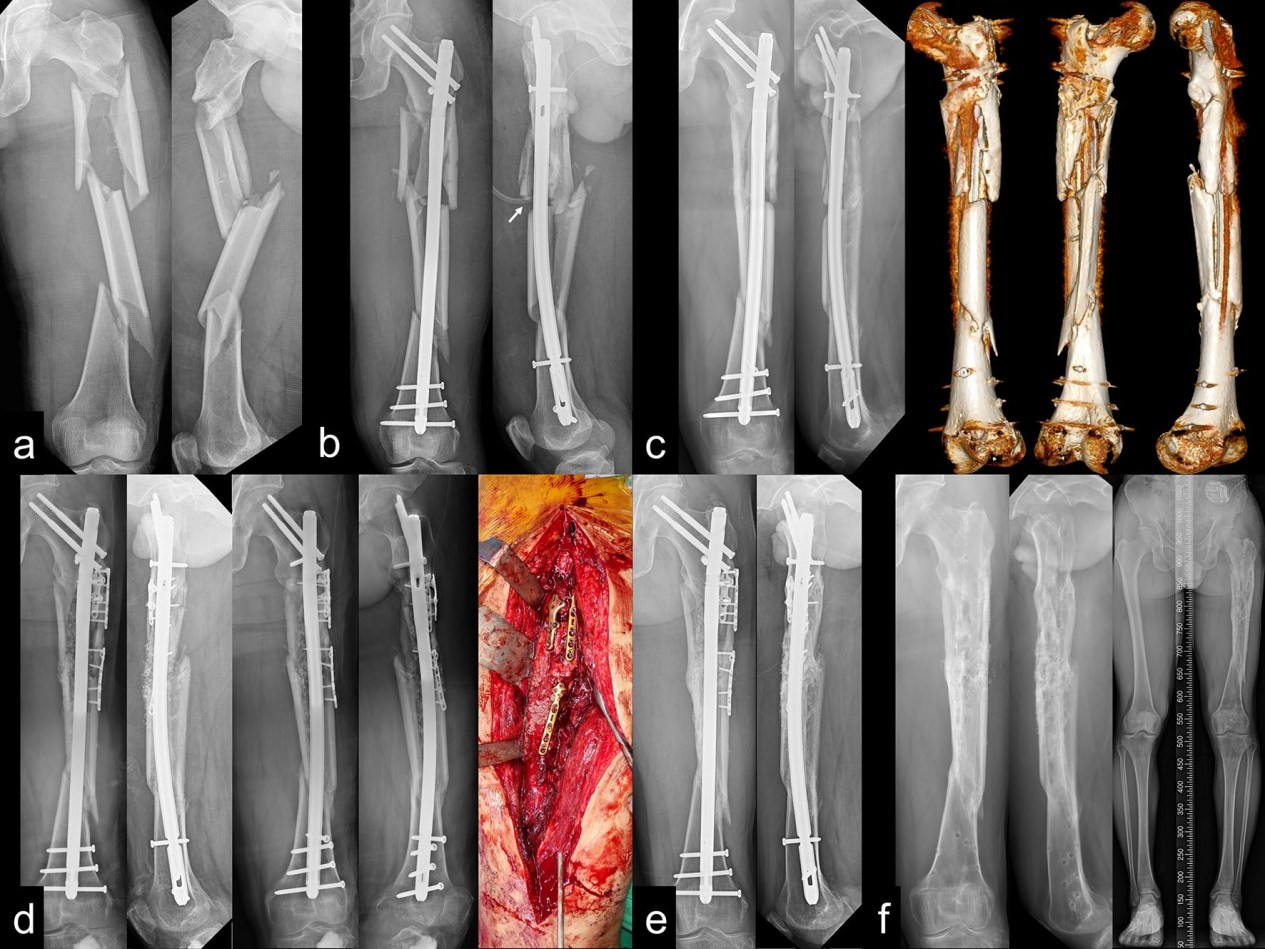
(**a**) A 51-year-old male patient was diagnosed with a femoral shaft fragmentary segmental fracture (AO/OTA 32C3) on the left side. (**b**) Reamed antegrade intra-medullary femoral nailing was performed using two reconstruction proximal interlocking screws, one proximal blocking screw and four distal interlocking screws. The exposed nail length was measured as 28 mm in immediate post-operative radiographs. (**c**) Nine months after surgery, the patient developed non-union. Three-dimensional reconstruction of computed tomography images showed the nail partially exposed owing to cortical bone defects where comminution was initially located. (**d**) On diagnosis of non-union, the patient underwent osteotomy and repositioning of vitalised cortical bone fixed with small fragment-specific plates, followed by autogenous and bone substitute grafts to fill the cortical bone defect. (**e**) Non-union completely healed 13 months following revision surgery. (**f**) X-ray image showing a fully consolidated and united femoral shaft fragmentary segmental fracture after implant removal surgery. There was no significant angular deformity or shortening with acceptable alignment.

Union was achieved in 37 patients (72.6%), and the mean time to union was 10.7 ± 4.8 months. Non-union was noted in 14 patients (27.4%) (classification: oligotrophic type, 10 patients; atrophic type, 4). Additional plating or exchange nailing combined with autogenous bone grafting was performed as revisional surgery for both non-union types^23–25^ (Fig. 3).

Comparison between the union and non-union groups revealed no significant differences in the demographic data (Table 1). Furthermore, when comparing the treatment-related characteristics, there were no significant differences in the number of proximal and distal interlocking screws, segmentation length, main third fragment length, distance between the main third fragment and intact shaft and width ratio from immediate post-operative radiographs between the groups. The exposed nail length in one cortex was significantly higher in the non-union group than in the union group (Table 2). Alignment and length were analyzed in all patients. Out of these, 30 patients (58.8%) had an acceptable alignment and length. However, 10 patients (19.6%) had a combined malalignment which includes axial malalignment and limb length discrepancy. Axial malalignment was observed in 16 patients, accounting for 31.4%. In the coronal plane, 8 patients had varus deformity, and 3 patients had valgus deformity. In the sagittal plane, 2 patients had flexion deformity, and 1 patient had extension deformity. Only 2 patients showed multiple plane deformities. Additionally, limb length discrepancy was found in 15 patients, accounting for 29.4%. Out of these 15 patients, 11 had shortening, and 4 had lengthening (Table 3).

**Table 1:** Demographic data of each group.

	Total	Non-union group	Union group	*p*-value
Number, n (%)	51	14 (27.4)	37 (72.6)	NA
Sex (male/female), n	43/8	14/0	29/8	0.08
Age (years), mean (SD)	40.1 (13.7)	33.7 (14.2)	42.5 (12.8)	0.054
BMI (kg/m^2^), mean (SD)	24.4 (3.1)	26.0 (3.9)	23.8 (2.6)	0.103
DM, n (%)	7	1 (7.1)	6 (16.2)	0.657
Smoking, n (%)	20	6 (42.9)	14 (37.8)	0.743
Side (right/left), n	51	18/19	6/8	0.712
Injury mechanism, n (%)				0.382
Slip over	1 (2)	1 (7.1)	0	
Fall from height	12 (23.5)	3 (21.4)	9 (24.3)	
MVA	37 (72.5)	10 (71.4)	27 (73)	
Sports	1 (2)	0	1 (2.7)	
Polytrauma, n (%)	43	11 (78.6)	32 (86.5)	0.07
Time to surgery (days), mean (SD)	5.45 (4.0)	5.3 (3.6)	5.9 (4.9)	0.791
Follow-up duration (months), mean (SD)	36.8 (17.0)	34.0 (13.3)	37.9 (15.6)	0.840
Open fracture, n (%)	7 (13.7)	1 (7.1)	6 (16.2)	0.08
Open fracture (subtype), n (%)				0.07
I	4	1 (7.1)	3 (8.1)	
II	2	0	2 (5.4)	
III	1	0	1 (2.7)	
Fracture location, n (%)				0.910
i (proximal dia-metaphyseal)	11 (21.5)	3 (21.4)	8 (21.6)	
j (pure diaphyseal)	27 (52.9)	8 (57.1)	19 (51.4)	
k (distal dia-metaphyseal)	13 (25.5)	3 (21.4)	10 (27.0)	
Number of fragments, mean (SD)	3.3 (1.4)	3.4 (1.2)	3.3 (1.4)	0.907
Time to union (months), mean (SD)	10.7 (4.8)	NA	10.7 (4.8)	NA

*BMI* body mass index, *DM* diabetes mellitus, *HTN* hypertension, *MVA* motor vehicle accident, *NA* not applicable.

**Table 2:** Surgical details of each group.

	Total	Nonunion (%)	Union (%)	*p*-value
Number, n (%)	51	14 (27.4)	37 (72.6)	
Implant, n (%)				0.910
A2FN	27 (50.9)	8 (57.2)	19 (51.4)	
FRN	13 (24.5)	3 (21.4)	10 (27.0)	
ZNN	11 (20.8)	3 (21.4)	8 (21.6)	
Number of proximal interlocking screws, mean (SD)	2.1 (0.5)	2.2 (0.6)	2.1 (0.4)	0.261
Number of distal interlocking screws, mean (SD)	3.2 (1.2)	3.7 (1.3)	3.0 (1.2)	0.065
Segmentation length (mm), mean (SD)	127.9 (46.6)	122.7 (49.7)	129.8 (46.0)	0.627
Main third fragment length (mm), mean (SD)	108.9 (34.1)	96.8 (33.0)	113.5 (33.6)	0.116
Distance of the main third fragment (mm), mean (SD)	16.8 (9.4)	17.5 (12.5)	16.6 (8.1)	0.748
Width ratio, mean (SD)	1.78 (0.53)	1.8 (0.7)	1.8 (0.4)	0.656
Exposed nail length (mm), mean (SD)	19.4 (26.9)	50.4 (36.7)	7.8 (6.7)	< 0.001

*A2FN* Expert Asian Femoral Nail, *FRN* Femoral Recon Nail, *ZNN* Zimmer Natural Nail.

**Table 3:** Radiologic outcomes.

	Total	Nonunion (%)	Union (%)	*p*-value
Number, n (%)	51	14(27.4)	37(72.6)	
Acceptable alignment (Axial and Length)	30(58.8)	8(57.1)	22(59.5)	0.563
Combined Malalignment (Axial and Length)	10(19.6)	3(21.4)	7(18.9)	0.561
Axial malalignment, n (%)	16(31.4)	4(28.6)	12(32.4)	0.537
Varus	8(15.7)	2(14.3)	6(16.2)	
Valgus	3(6.9)	1(7.1)	2(5.4)	
Flexion	2(3.9)	1(7.1)	1(2.7)	
Extension	1(2.0)	0(0)	1(2.7)	
Multiple plane	2(3.9)	0(0)	2(5.4)	
Limb length discrepancy, n (%)	15(29.4)	4(28.6)	11(29.7)	0.611
Shortening	11(21.6)	3(21.4)	8(21.6)	
Lengthening	4(7.8)	1(7.1)	3(8.1)	

**Figure 4:**
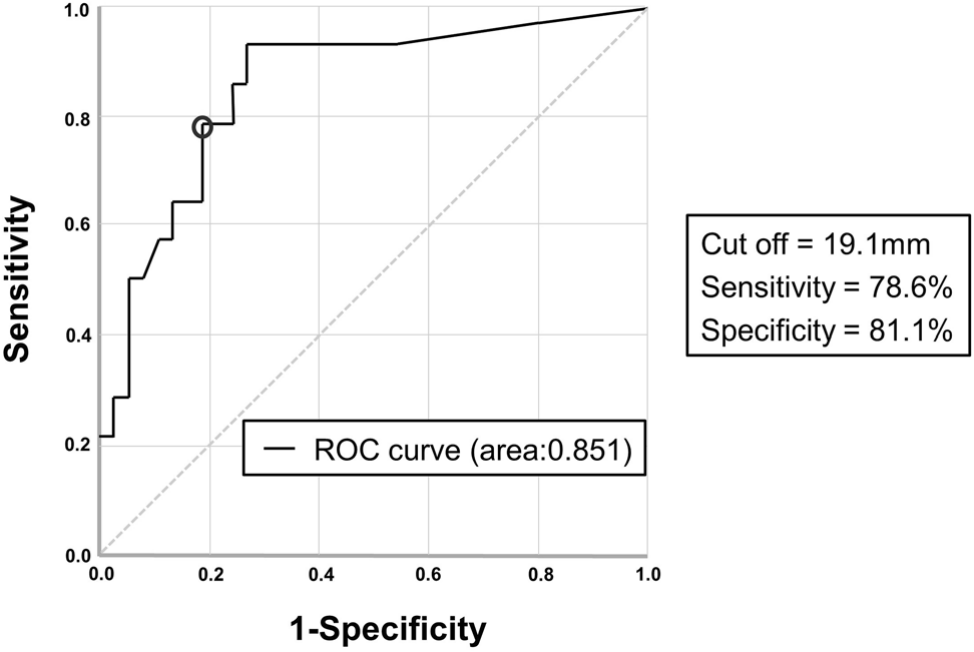
Receiver operating characteristic curve analysis to determine the cut-off of the exposed nail length in one cortex. A value greater than 19.1 mm had a sensitivity of 0.786 and specificity of 0.811 for predicting the occurrence of non-union.

In the multivariate analysis, only exposed nail length was associated with non-union (odds ratio: 1.599, *p* = 0.003) (Table 4). To predict the non-union risk, the exposed nail length cut-off was determined (Fig. 4), and value of 19.1 mm had a sensitivity of 0.786 and specificity of 0.811, indicating that it was a reliable predictor. The area under the curve was 0.851 (95% CI 0.729–0.974), indicating good diagnostic accuracy (*p* < 0.001). Cohen’s kappa values of the variables for inter-observer/intra-observer reliability were 0.72–0.83.

**Table 4:** Logistic regression analysis of non-union risk factors for fragmentary segmental fractures of the femur shaft.

Variable	Univariate		Multivariate	
	Odds ratio (95% CI)	*p*-value	Odds ratio (95% CI)	*p*-value
Polytrauma	0.870 (0.713–1.063)	0.093	-	
Time to surgery	0.840 (0.675–1.046)	0.142	-	
Number of distal interlocking screws	1.008 (0.976–1.041)	0.116	-	
Width ratio	2.595 (0.300–22.458)	0.144	-	
Exposed nail length	1.626 (1.055–2.593)	0.023	1.599 (1.084–2.127)	0.003

*CI* confidence interval.

## Discussion

The current study evaluated the radiographic outcomes of reamed antegrade intra-medullary nailing in treating femoral shaft fragmentary segmental fractures (AO/OTA 32C3) with extremely low incidence and the risk factors associated with non-union.

In our results, the non-union rate was 27.4% (14/51). In general, the non-union rate of femoral shaft fractures is approximately 5–7%^25–28^. However, since multiple factors influence fracture healing, non-union rates have been reported to vary widely in various studies. Although there is a lack of research on the outcome of femoral shaft fragmentary segmental fractures, our results show a higher non-union rate than the overall non-union rate of femoral shaft segmental fractures (AO/OTA 32C) treated with intra-medullary nailing. Kempf et al.^29^, Wiss et al.^30^ and Karadimas et al.^3^ reported non-union rates of 10% (5/52), 2% (1/33) and 3.8% (5/131), respectively. Recently, Kook et al.^31^ reported a non-union rate of 15.8% (6/38) for intact segmental femoral shaft fractures treated with intra-medullary nail fixation (AO/OTA 32C2). The findings of those researches are relatively lower than what we have observed.

Moreover, it was found that only 58.8% of patients had acceptable axial alignment and length from the current study. Additionally, 31.4% of patients had axial malalignment, and 29.4% had limb length discrepancy. The malalignment rate was 41.2% overall. So far, the reported incidence of malalignment during femoral shaft fracture treatment with nailing ranges from 0 to 37%^32–35^. Ricci et al.^21^ reported that 9% of 355 femur shaft fractures were axially malaligned, while the incidence of axial malalignment was higher for proximal third femoral shaft fractures (30%) and highly comminuted fractures (17%). According to Winquist and Hansen^36^, a higher degree of fracture comminution resulted in a greater incidence of LLD with Winquist types III and IV displaying 14.8% and 22.8%, respectively. Vaidya et al.^22^ and Gheraibeh et al.^20^ reported a 22% and 21.4% leg length discrepancy, respectively, after intramedullary nailing for femoral shaft fractures. Our cohort only included patients with femoral shaft segmental fractures (AO/OTA 32C3), which may lead to a slightly higher incidence compared to previous studies.

A possible explanation for our high non-union rate is that femoral shaft fragmentary segmental fractures (AO/OTA 32C3) are usually caused by severe soft tissue damage resulting from high-energy trauma. An open fracture is a well-known risk factor for non-union, and its non-union rate is more than twice that of a closed fracture^24^. However, in this study, there was no statistical significance. As almost all open fracture patients had Gustilo–Anderson type I or II injuries, which are less destructive, the damage to the soft tissue structure and biology was not severe. Moreover, as the muscular envelope is more abundant around the femur than around other long bones, general wound management, including debridement and primary repair, was feasible in most cases.

Apart from the increase in non-union rate due to damaged soft tissue envelope, treating a femoral shaft segmental fracture with IM nailing is extremely tricky in terms of fracture reduction. The presence of multiple fragments with no contact between the main fragments makes it challenging to achieve appropriate axial, rotational and length alignments when compared with simple (AO/OTA 32A) or wedge fractures (AO/OTA 32B), and intact segmental fractures (AO/OTA 32C2). In particular, assessing functional alignment is more difficult within the narrow c-arm field of view. These limitations can lead axial malalignment and limb length discrepancy. In the process of closed reduction during the operation, one end of the bony fragment is unsupported and floating. Thus, a percutaneous technique is usually required as a simple closed manoeuvre is not sufficient. Moreover, fragmentary segments may be incompletely reamed during intra-medullary reaming, leading to deviation from anatomical alignment. Furthermore, during surgery for AO/OTA 32C3 fractures, significant resistance may be encountered while inserting a larger-diameter nail, which can result in reduction loss of segmental or wedge fragments. Millar et al.^37^ recommended a minimum nail fit of 70% at the isthmus and ideally ≥ 90% to avoid reoperation from aseptic and hypertrophic femoral non-union on radiological analysis. Therefore, small-diameter nails after insufficient reaming are not recommended for stable fixation.

Several studies have reported on and analysed the third fragment, which is the intact wedge fragment associated with femoral shaft fractures (AO/OTA 32B2). Vincenti et al.^15^ found that the third fragment size (40-mm cut-off) was a key risk factor for non-union in femoral shaft wedge fractures (AO/OTA 32B2) treated with intra-medullary nailing. Additionally, they reported that displacement of the main third fragment (12-mm cut-off) was a key risk factor for delayed union. Lee et al.^38^ reported that both the size (80-mm cut-off) and displacement (proximal, 20-mm cut-off; distal, 10-mm cut-off) of the third fragment were risk factors for non-union and concluded that the fragment displacement angle had a greater influence on the union rate than the size. Conversely, Hamahashi et al.^39^ reported that displacement of the third fragment was the only risk factor for delayed union, and Lin et al.^40^ demonstrated that the fragment width ratio could predict non-union for femoral shaft fractures with the third fragment (0.55 cut-off). These studies analysed the characteristics of the third fragment that can affect fracture union in AO/OTA 32B2 fractures according to the importance of fracture morphology and reduction.

The third fragment has an intact wedge shape that can be analysed and measured. However, in fragmentary segmental femoral fractures (AO/OTA 32C3), the main third fragment cannot be identified owing to multiple fragments and lack of standardisation. Therefore, other parameters for risk factor evaluation are needed. The concept of ‘exposed nail length’ was introduced in this study, which suggests that the characteristics of the third fragment can alter the local environment, similar to the hypothesis of previous studies regarding AO/OTA 32B2 fractures. It is partially similar to the residual fracture gaps in simple fractures, which are caused by distraction or mal-reduction of angulation and rotation. However, there is a lack of compatibility in terms of strain. According to Parren’s strain theory, fracture gaps in simple fractures result in higher strain compared with comminuted fractures.

In this study, only exposed nail length was associated with non-union in the multivariate analysis. In contrast to previous findings^24,26^, other demographic variables, which are well-known risk factors for non-union, did not show significance in our study. An exposed nail length cut-off of 19.1 mm was identified. This finding holds notable importance regarding the non-union rate and could be utilized as valuable evaluative criteria for supplementary interventions, such as cerclage wiring and augmentation plating. Although their effectiveness is unclear and debatable, these procedures could be considerable as an alternative to reduce non-union risk. Obviously, it is important to give careful consideration to the soft tissue surrounding the fracture. Anteroposterior, lateral and oblique views are typically used to detect bone defects in a clinical setting, and thus, the defects may not be visible depending on the projection angle of the c-arm. Furthermore, theoretically, if the fracture involves severe comminution, the exposed nail length will inevitably increase. In actual clinical practice, we suggest using twice the nail diameter instead of 19.1 mm.

This study has several limitations. First, the study design was retrospective, and the sample size of non-union patients was small. However, it is challenging to enrol a high number of patients as femoral shaft fragmentary segmental fractures are relatively rare. Second, 13 patients were excluded from the analysis for different reasons, such as death and loss to follow-up, which could introduce selection bias. Third, various radiological parameters^15,38,39^ thought to affect fracture healing are not standard or representative of non-union. We attempted to identify significant radiologically measurable parameters that could describe and reflect the morphology of fragmentary segmental femoral fractures (AO/OTA 32C3). Fourth, post-operative reduction states, such as angular and rotational alignment and limb length restoration, were not evaluated, although these can affect the non-union rate. In the subtrochanteric area, varus alignment is a well-known risk factor. Fifth, if the cortical deficiency is extremely large on only one side of the cortex, it can affect the overall result. In this study, no patient had cortical defects on only one side with three intact cortices. Moreover, in the clinical setting, it is difficult to estimate the actual length. Thus, we recommend using the ratio of exposed nail length to nail diameter, with 19.1 mm being approximately double the nail diameter.

To our knowledge, this is the first study to evaluate the risk factors for non-union of femoral shaft fragmentary segmental fractures after reamed antegrade intra-medullary nailing and report radiographic outcomes.

## Conclusion

Reamed antegrade intra-medullary nailing for femoral shaft fragmentary segmental fractures (AO/OTA 32C3) showed high rates of non-union (27%) and malalignment (41%). An exposed nail length of > 19.1 mm was associated with non-union. Therefore, patients who underwent intramedullary nailing with an exposed nail length greater than 19.1 mm or about twice the nail diameter should be cautioned of the potential non-union.

## Data Availability

The datasets generated during and/or analysed during the current study are available from the corresponding author on reasonable request.
